# Transplacental transfer of Lassa IgG antibodies in pregnant women in Southern Nigeria: A prospective hospital-based cohort study

**DOI:** 10.1371/journal.pntd.0011209

**Published:** 2023-04-13

**Authors:** Nzelle Delphine Kayem, Sylvanus Okogbenin, Joseph Okoeguale, Joseph Eigbefoh, Joseph Ikheloa, Reuben Eifediyi, Xavier Enodiana, Olugbenga Emmanuel Olorogbogo, Isoken Aikpokpo, Yemisi Ighodalo, Thomas Olokor, George Odigie, Lyndsey Castle, Sophie Duraffour, Lisa Oestereich, Prabin Dahal, Proochista Ariana, Stephan Gunther, Peter Horby

**Affiliations:** 1 Nuffield Department of Medicine, University of Oxford, Oxford, United Kingdom; 2 Department of Obstetrics and Gynaecology, Irrua Specialist Teaching Hospital, Irrua, Nigeria; 3 Institute of Lassa fever Research and Control, Irrua Specialist Teaching Hospital, Irrua, Nigeria; 4 Bernhard-Nocht-Institute for Tropical Medicine, Hamburg, Germany; Universidade do Estado do Rio de Janeiro, BRAZIL

## Abstract

**Background:**

Evidence from previous studies suggest that Lassa fever, a viral haemorrhagic fever endemic to West Africa has high case fatalities, particularly in pregnancy. While there have been remarkable innovations in vaccine development, with some Lassa vaccines undergoing early clinical trials. An understanding of Lassa antibody kinetics and immune responses will support vaccine design and development. However, there is currently no evidence on the antibody kinetics of Lassa (LASV) in pregnancy. Our study sought to estimate the efficiency of transplacental transfer of LASV IgG antibodies from the mother to the child.

**Methodology/Principal findings:**

The study made use of data from a prospective hospital-based cohort of pregnant women enrolled at the antenatal clinic and followed up at delivery between February and December 2019. Blood samples from mother-child pairs were evaluated for antibodies against Lassa virus. The study demonstrates a transplacental transfer of LASV IgG of 75.3% [60.0–94.0%], with a significant positive correlation between maternal and cord concentrations and a good level of agreement. The study also suggests that transfer may be more variable in women with ‘*de novo’* antibodies compared to those with pre-existing antibodies.

**Conclusions/Significance:**

The study shows that maternal antibody levels play an important role in determining transfer efficiency of Lassa antibodies to the new-born; and while the evidence is preliminary, the study also suggests that transfer efficiency may be less stable in acute or recent infection, as such timing of vaccination before pregnancy, that is in women of childbearing age may be more appropriate for protection of both pregnant women and their neonates.

## Author summary

Lassa fever, an epidemic-prone viral infection which frequently occurs in West African countries has a high mortality in pregnant women and their new-borns. Vaccination is an ideal method for preventing infection and while there are currently no licensed vaccines for Lassa fever, there have been marked innovations in Lassa vaccine development with some vaccines currently in early trials. Vaccine development requires background information on disease prevalence, incidence, and antibody kinetics. Our study sought to understand the kinetics of Lassa antibodies in pregnant women and their new-borns. This is the first study to evaluate the transfer of Lassa antibodies between the mother and child. We found that maternal antibody levels play an important role in determining how well antibodies are transferred from the mother to the child and may be influenced by the time when antibodies developed, with transfer of antibodies being less stable in those with recent infection. This suggest that for protection of both pregnant women and their neonates, it may be more appropriate to vaccinate women before they become pregnant, that is women of child-bearing age. While, the evidence is not conclusive, it is an important first step to understanding the kinetics of Lassa antibodies.

## Introduction

Lassa fever, a viral haemorrhagic fever caused by Lassa virus, is classified by the World Health Organization (WHO) as a priority pathogen [1,2]. It is thought to be endemic to West Africa where it puts an estimated 37.7 million people in 14 West African countries at risk of infection [3], with over 100,000 to 500,000 infections [4,5], and approximately 5000 deaths annually [4]. In pregnancy, a recent meta-analysis suggests that pregnant women who are diagnosed with acute Lassa fever are three times more likely to die than their non-pregnant counterparts with an estimated foetal loss of 61.5% [6].

In recent years, there has been a marked drive for development of countermeasures against priority pathogens like Lassa virus [1,2]. Lassa fever has one of the most advanced development platforms with six vaccines funded by the Coalition for Epidemic Preparedness Innovations (CEPI) [7]. Two of these vaccines are undergoing phase I trials; a DNA vaccine (NCT03805984) and a Measles virus vectored vaccine (NCT04055454)) [8,9]. Inovio’s DNA vaccine is the most advanced and has completed Phase IB trials in Ghana [10]. Inclusion of pregnant women in future vaccine trials would facilitate evaluation of vaccine efficacy and effectiveness in the context of pregnancy and provide evidence for vaccine program implementation.

Neonates depend on passive transfer of maternal immunoglobulin G (IgG) antibodies for protection against many neonatal infections, and transplacental transfer is the major route by which maternal antibodies are transmitted to the foetus [11–13]. Most studies on Lassa-specific antibody patterns reflect the dynamics of Lassa virus (LASV) antibody activity in adults [14,15], and to the best of our knowledge, there are no studies that have compared maternal and neonatal LASV antibody levels or analysed potential factors that may impact the transplacental transfer of Lassa-specific antibodies. A better understanding of Lassa antibody kinetics and immune responses will support vaccine design and development.

We sought to estimate the efficiency of placental transfer of LASV IgG antibodies and assess factors associated with transplacental transfer of LASV IgG and changes in cord LASV IgG concentrations.

## Methods

### Ethics statement

Ethical approval was obtained from the Oxford Tropical Research Ethics Committee (OxTREC) of the University of Oxford (OxTREC reference No.: 49–18), and the Irrua Specialist Teaching Hospital Research Ethics Committee (ISTH REC), Edo State, Nigeria (HREC Approval No.: NHREC/29/03/2017). Written informed consent was obtained from all participants.

### Study population and study procedures

Lassa fever occurs throughout the year in Nigeria [16,17], with three states in Southern Nigeria frequently reporting high numbers of confirmed cases, and these are Edo, Ondo and Ebonyi state [16–18]. Studies from southern Nigeria suggest that Edo state alone accounts for over 60% of all confirmed cases in the south of Nigeria [16].

The study was conducted at the maternity or labour wards of Irrua Specialist Teaching Hospital (ISTH) and two health centres (Usugbenu primary centre and Eromosele medical centre) in Edo state, Southern Nigeria. These centres were chosen because they frequently refer patients to ISTH and were willing to participate in research studies. ISTH is a university teaching hospital and a centre of excellence for Lassa fever management in Nigeria [16].

The data used for this study were generated as part of a prospective hospital-based cohort with pregnant women enrolled at the antenatal clinic and followed up at delivery to assess seroconversion and seroprevalence. Participants for the cohort study were recruited from 12^th^ February 2019 to 4^th^ May 2019 and followed up until the last delivery which occurred on 20^th^ December 2019. Pregnant women were enrolled into the study if they were 15 years and above, attending ANC at one of the selected sites, and consented to participate. A structured questionnaire was administered within 48 hours of delivery to collect clinical and demographic information. Data were later entered and managed using REDCap (Research Electronic Data Capture) electronic data capture tools [19]. Clinical data such as data on malaria, HIV, diabetes, and hypertension were self-reported and verified with data from patient-held antenatal clinic books and/or patient hospital records where available. Mothers attending the facilities were routinely given intermittent preventive treatment with sulfadoxine pyrimethamine (iPTp-SP) as per Nigerian guidelines. Gestational age at birth was estimated using the Ballard Score [20]. Gestational age below 37 weeks was defined as prematurity, and birthweight below 2.5Kg was defined as low birth weight.

### Sample collection and serology

Whole blood samples (2 – 5mL) were collected from mothers within 48 hours of delivery, while umbilical cord blood (2.5mL) was taken directly from the placental end of the cord immediately after delivery. Samples were centrifuged, and serum was separated into cryotubes under sterile conditions; 0.1–0.2mL of serum aliquots were labelled and stored at –20°C until antibody ELISA (enzyme-linked immunosorbent assay) was performed.

There is currently no gold standard or reference test for definitive laboratory diagnosis or serological testing of Lassa fever [21,22]. The assay used in this study was one developed and previously used in this region showing a high specificity of 95–100% [23]. LASV nucleoprotein (NP) specific IgG antibodies were detected using BLACKBOX LASV IgG ELISA (Diagnostics Development Laboratory, Bernhard-Nocht Institute for Tropical Medicine, Hamburg, Germany) [23]. Given that, the transfer efficiency of LASV IgG is unknown, tetanus toxoid (TT) antigen (Virotech Diagnostics GmbH, Rüsselsheim, Germany) was used as a comparator (control) for transfer efficiency because mothers in Nigeria are routinely immunised against tetanus during pregnancy. Moreover, transplacental transfer of TT antibodies has been previously evaluated in numerous studies [24–28].

LASV IgG (BLACKBOX LASV IgG ELISA Diagnostics Development Laboratory, Bernhard-Nocht Institute for Tropical Medicine, Hamburg, Germany) and Tetanus IgG ELISA (Virotech Diagnostics GmbH, Rüsselsheim, Germany) were performed following the manufacturer’s instructions.

The BLACKBOX LASV IgG ELISA is an immune-complex binding ELISA used for detection of LASV IgG expressed as an index value (IV), calculated using optical density (OD) values. An index value of 1.1 or above (≥1.1) was considered positive for past or current infection, 0.9 or below (≤0.9) was negative, and values between 0.9 and 1.1 were equivocal. The Virotech TT IgG ELISA was an indirect quantitative ELISA with titres expressed following the WHO standard in International Units (IU/ml) based on extrapolation from a standard curve plotted on a semi logarithm scale. A TT IgG titre of over 0.1IU/ml was considered seropositive and indicative of vaccine protection. Instructions for use are readily available from the manufacturers at http://www.virotechdiagnostics.com/en/products.html.

### Statistical analysis

Data were analysed using R statistical software version 4.0.2 [29]. Logarithms of maternal and cord antibody levels were used to calculate the geometric mean antibody titres for TT IgG and the geometric mean concentrations for LASV IgG with the corresponding 95% confidence intervals. Placental transfer was measured as the ratio of the level of specific antibodies in cord blood to that in the maternal blood, that is, the cord maternal ratio (CMR).

The correlation between the levels of maternal LASV and TT IgG antibodies to those in the cord blood was calculated using the Spearman correlation coefficient, as it is less sensitive to outliers than the Pearson correlation coefficient. The level of agreement between maternal and cord concentrations was evaluated using Bland-Altman (B-A) plots [30]. Agreement was assessed because while correlation allows an evaluation of relationships between two variables [31], it does not address concordance between the maternal and cord concentrations, and paired tests assess for the overall mean differences and fail to account for individual differences [31]. The B-A plot provides a quantitative estimate of the relationship [31] between maternal and cord concentrations. B-A plots allow for a comparison of agreement between two quantitative measurements by constructing limits of agreement [32]. Conventionally, B-A plots are such that 95% of data points fall within the limits of agreement [33]. The mean difference should normally be as close to zero as possible, and there should generally be no trend in the difference as the average increases or decreases [32]. Additionally, the variance in the differences between the two measurements should be consistent [32]. For this study, given that there are no data on clinical limits of agreement, we decided pragmatically, that the prespecified clinical limits of agreement would be two standard deviations (+/- 2SD). For easy visualisation and to facilitate interpretation of the patterns in TT IgG, we log-transformed the data for all TT plots as this helped reduce skewness in the data. For LASV IgG, log transformation did not improve skewness as such data were not transformed for analysis.

Linear regression analysis was used to assess the effect of different maternal and new-born characteristics on the cord maternal ratio (CMR) and cord concentrations for LASV IgG and TT IgG. Risk factors for LASV IgG seronegativity (index value ≤0.9) in children born to LASV IgG seropositive mothers were evaluated using Fisher’s exact test.

For all statistical analyses, a two-sided p-value of ≤ 0.05 was considered statistically significant. However, for multiple comparisons, the Bonferroni correction was applied. For all regressions, a univariable regression was first performed and only marginally significant variables (P< 0.25) from the univariable regression were considered in the multivariable regression [34], and there were no imputations for missing data; consequently, denominators vary by response. Collinear terms for the study were age and parity; gestational age at birth and birth weight; and fever and malaria during pregnancy. Thus, if both collinear terms were significant in the univariate model, only age, malaria, or gestational age at birth were then included in the multivariable model as these terms were considered to be more biologically relevant for antibody changes.

## Results

A total of 240 pregnant women were enrolled but complete serological data were available for 172 mother-child pairs. Of these 170 had singleton deliveries, and two were multiple pregnancies. Our analysis only included the singleton deliveries. Amongst the singleton deliveries, 77 mothers were LASV IgG seropositive at delivery with an estimated seroprevalence of 45.7% [95% CI: 38.4–53.1%]. Consequently, to evaluate transfer efficiency of LASV IgG antibodies, there are a total of 77 seropositive mother-child pairs, while for tetanus toxoid IgG antibodies, all 170 mother-child pairs were included in the analysis.

### Baseline characteristics

Detailed characteristics of mothers and neonates are summarised in Table 1. Overall, the mean maternal age was 31.6 ± 4.8 years [range: 20.0–46.0 years], while the mean maternal age amongst seropositive mothers was 31.6 ± 5.3 years [range: 20.0–41.0 years]. The mean gestational age at birth (based on Ballard’s score) was 39.0 ± 2.0 weeks [range: 29.0–43.0 weeks], and amongst seropositive mothers, the mean gestational age was 39.0 ± 1.8 weeks [range: 34.0–43.0 weeks]. Sixty-seven (30·4%) women reported malaria, and use of artemisinin combination therapy (Coartem) was reported in two mothers while quinine was recorded in 59 mothers. Thirty-four LASV IgG seropositive mothers reported malaria, and all had received quinine. Eight (4.7%) of HIV-infected mothers were retained in the study, seven of whom were LASV IgG seropositive. Four (2.4%) women with a history of prior Lassa fever were retained, of these, two were LASV IgG seropositive.

**Table 1:** General characteristics of mothers-child pairs.

Maternal characteristics at delivery	N (%)	LASV IgG Seropositive (%)
Total	170	77
Maternal age (years)		
Mean ± SD [range]	31·6 ± 4·8 [20–46]	31·6 ± 5·3 [20–41]
≤ 25	21 (12·4)	11 (14·3)
26–30	51 (30·0)	22 (28·6)
31–35	72 (42·4)	30 (38·9)
> 36	26 (15·3)	14 (18·2)
Lives in rural area	94 (55·3)	38 (49·3)
Education		
Primary	8 (4·7)	6 (7·8)
Secondary	33 (19·4)	20 (25·9)
Post—secondary	129(75·8)	51 (66·2)
Parity		
0	35(20·6)	17 (22·1)
1–2	72(42·4)	29 (37·7)
≥ 3	63 (37·0)	31 (40·3)
Gestational age at baseline (weeks)^a^		
Median (IQR)	24 [18–31]	23 [17–29]
≤ 13	20 (12.3)	10 (13.5)
14–27	85 (51.8)	40 (54.1)
>28	59 (35.9)	24 (32.4)
Positive history of fever during pregnancy	34 (20·0)	17 (22·1)
Positive history of prior LF	4 (2·4)	2 (2·6)
Positive history of malaria during pregnancy	67 (30·4)	34 (44·2)
HIV—infected	8 (4·7)	7 (9·1)
Diabetes Mellitus	4 (2·4)	2 (2·6)
Gestational diabetes	2 (1·2)	0
Hypertension	5 (2·9)	3 (3·9)
Pregnancy-induced hypertension	4 (2·4)	3 (3·9)
Total number of TT vaccinations received		
≥ 2	148 (87·1)	64 (83·1)
< 2	22 (12·9)	13 (16·9)
Maternal LASV IgG GMC	—	4·35 [95% CI: 4·04–4·68]
Maternal TT IgG GMT	1·46 [95% CI: 1·25–1·71]	1·55 [95% CI: 1·20–2·00]
Protective level of TT mother		
No	2 (2·2)	1 (1·3)
Yes	168 (98·8)	76 (98·7)
**New-born characteristics**		
Gestational age at birth (weeks)		
Mean ± SD [range]	39·2 ± 2·0 [29–43]^b^	39·4 ± 1·8 [34–43]^a^
≥ 37	139 (91·4)	65 (91·5)
< 37	13 (8·6)	6 (8·5)
Birthweight (Kg)		
Mean ± SD [range]	3·2 ± 0·5 [0·9–4·7]	3·2 ± 0·5 [2·1–4·3]
≥ 2·5	159 (93·5)	71 (92·2)
< 2·5	11 (6·5)	6 (7·8)
LASV IgG median CMR	—	0·753 [95% CI: 0·60–0·94]
Cord LASV IgG GMC	—	3·28 [95% CI: 2·62–4·09]
Cord TT IgG GMT	1·43 [95% CI: 1·24–1·67]	1·44 [95% CI: 1·13–1·83]
TT IgG median CMR	0·991 [95% CI: 0·88–1·11]	0·919 [95% CI: 0·78–1·09]
Protective level of TT neonate^c^		
No	2 (2·6)	2 (2·7)
Yes	166 (98·8)	73 (97·3)

**Note:** Data presented as n (%), median (IQR) or mean ± SD [range], as appropriate, where n is the number of mothers or neonates with a characteristic. CI- confidence interval; CMR- cord maternal ratio; GMC- geometric mean concentration; GMT-geometric mean titre; IgG- immunoglobulin G; IQR- interquartile range; LF- Lassa fever; LASV- Lassa virus; N- total number of pregnant women retained in the study; SD- standard deviation; TT- tetanus toxoid.

^a^ Data missing for 6 women.

^b^ Data missing for 18 women.

^c^ Data missing for 2 neonates.

Of the 77 mothers who were LASV IgG seropositive, 22 had seroconverted and 55 had been positive at both first (enrolment) and second blood draw (delivery); we classified these mothers as seropositive from baseline or women with pre-existing antibodies. Seroconversion was defined as a participant who was: seronegative for LASV IgG at baseline (index value ≤0.9); became seropositive at delivery (IgG index value ≥1.1); was in the study for at least 30 days, (that is the interval between the first maternal sample and the second maternal sample was ≥30 days); and had a change in the index value of four-fold or more.

A lower limit of 30 days was chosen because studies suggest that LASV IgG has a mean time to first detection of 25.6 ± 3 days after symptom onset [35]. A four-fold rise in the index value was used because a four-fold increase in antibody titre is conventional for an antibody change defining infection and seroconversion [36,37]. A fold change was calculated as the ratio of antibody concentration at delivery to that at enrolment. Characteristics of mothers and neonates based on maternal LASV IgG serostatus are summarised in S1 Table.

### Transplacental transfer of LASV and TT specific IgG antibodies

The geometric mean concentration of LASV IgG in mothers was 4.35 IV [95% CI: 4.04–4.68 IV] and in neonates was 3.27 IV [95% CI: 2.62–4.09 IV] while the geometric mean titre of TT IgG in mothers was 1.46 IU/ml [95% CI: 1.25–1.71 IU/ml] and in neonates was 1.44 IU/ml [95% CI: 1.24–1.67 IU/ml]. The median cord maternal ratio (CMR) for LASV IgG was 0.753 [95% CI: 0.60–0.94] and for TT IgG was 0.991 [95% CI: 0.88–1.11], this means the percent transfer for LASV, and TT IgG antibodies was 75.3% and 99.1% respectively. The relative maternal and cord geometric mean concentrations for different maternal characteristics with the corresponding median CMR are summarised in S2 and S3 Tables.

### Comparison of LASV and TT specific IgG levels in mothers and their neonates

**Fig 1:**
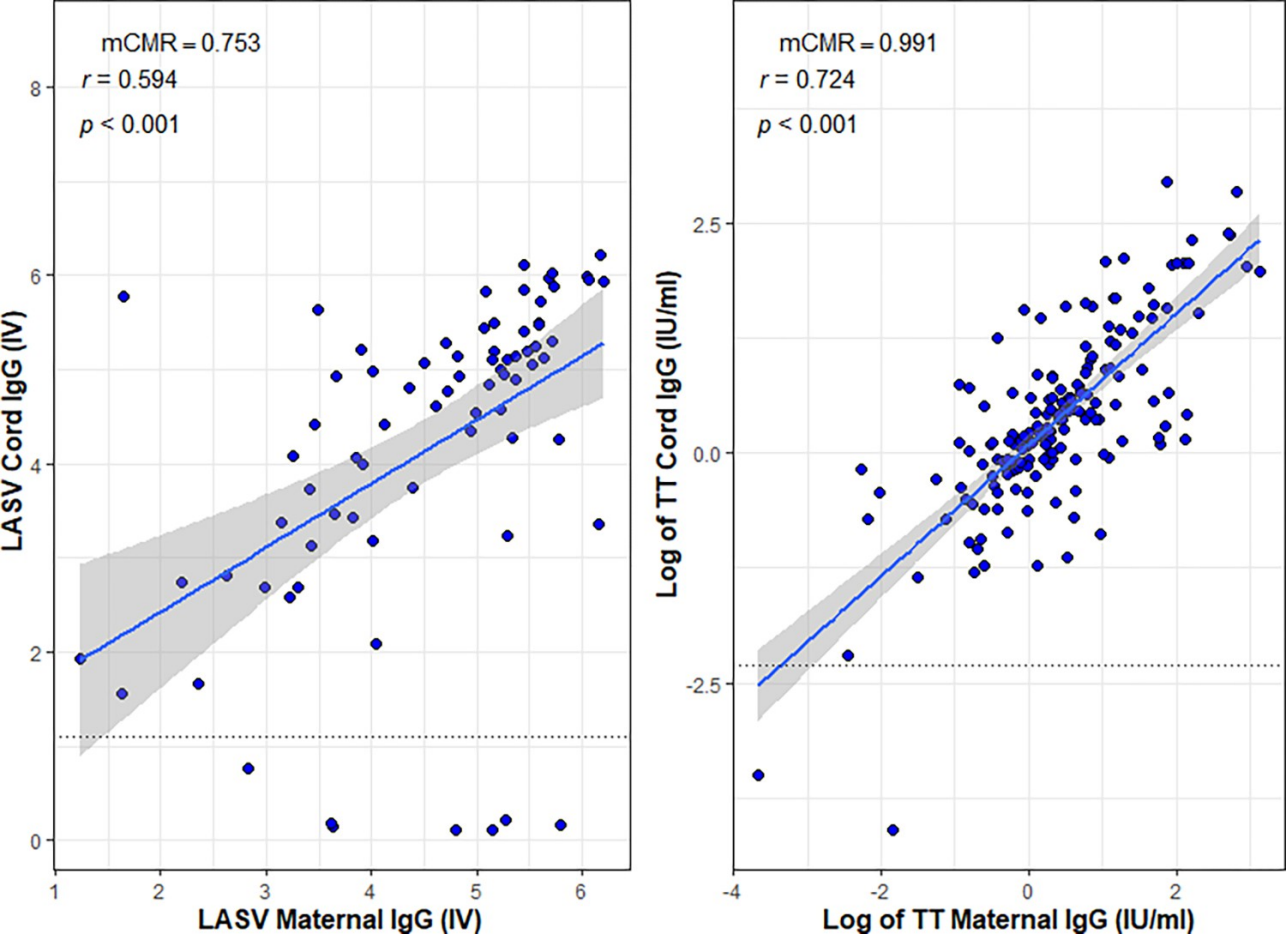
Correlation between maternal and cord Lassa and TT IgG antibodies assessed by Spearman’s correlation (r). Note: IV- index value; mCMR- median cord maternal ratio; black dotted horizontal line is line of seropositivity (LASV = 1.1, TT = 0.1IU/mL); blue line is the regression line; grey shaded are the 95% confidence interval fitted to the regression line.

An evaluation of the strength and direction of the relationship between maternal and neonatal levels showed a significant positive correlation between levels of maternal and cord antibodies for both LASV IgG (r = 0.594, p < 0.001), and TT IgG (r = 0.724, p<0.001), Fig 1.

**Fig 2:**
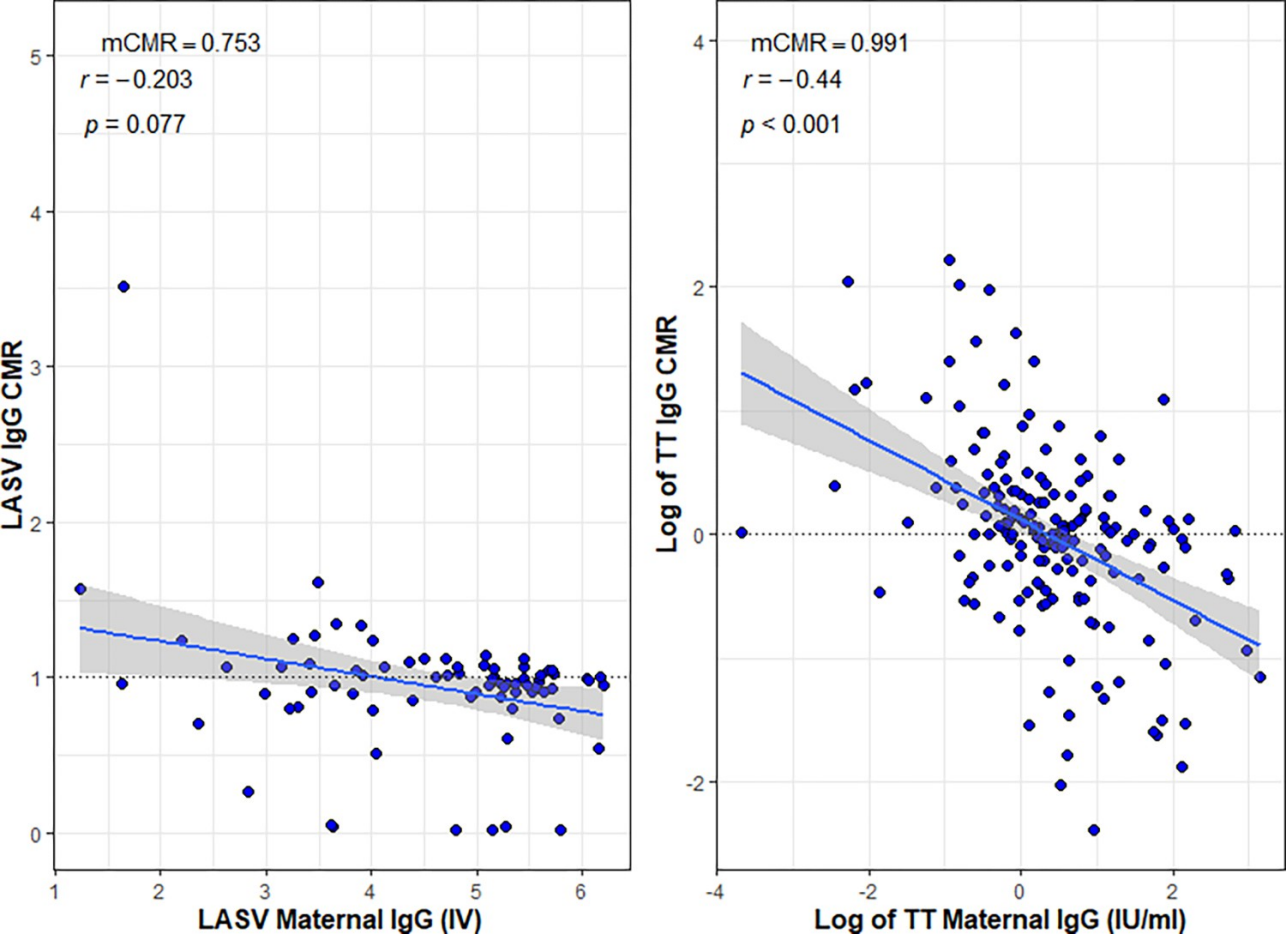
Correlation plots between cord maternal ratio and maternal LASV and TT IgG concentrations assessed by Spearman’s correlation (r). Note: mCMR- median cord maternal ratio; IV- index value, an arbitrary antibody concentration unit based on manufacturer’s guide; the dotted black horizontal line is the line of efficient transfer (CMR = 1); Log- natural logarithm; blue line is the regression line; grey shaded are the 95% confidence interval fitted to the regression line.

We also assessed the correlation between cord maternal ratio (CMR) and maternal IgG concentrations. TT IgG showed a significant negative correlation (r = -0.440, p<0.001), whereas the correlation between maternal LASV IgG and cord maternal ratio was not significant, (r = -0.203, p = 0.077), Fig 2.

**Fig 3:**
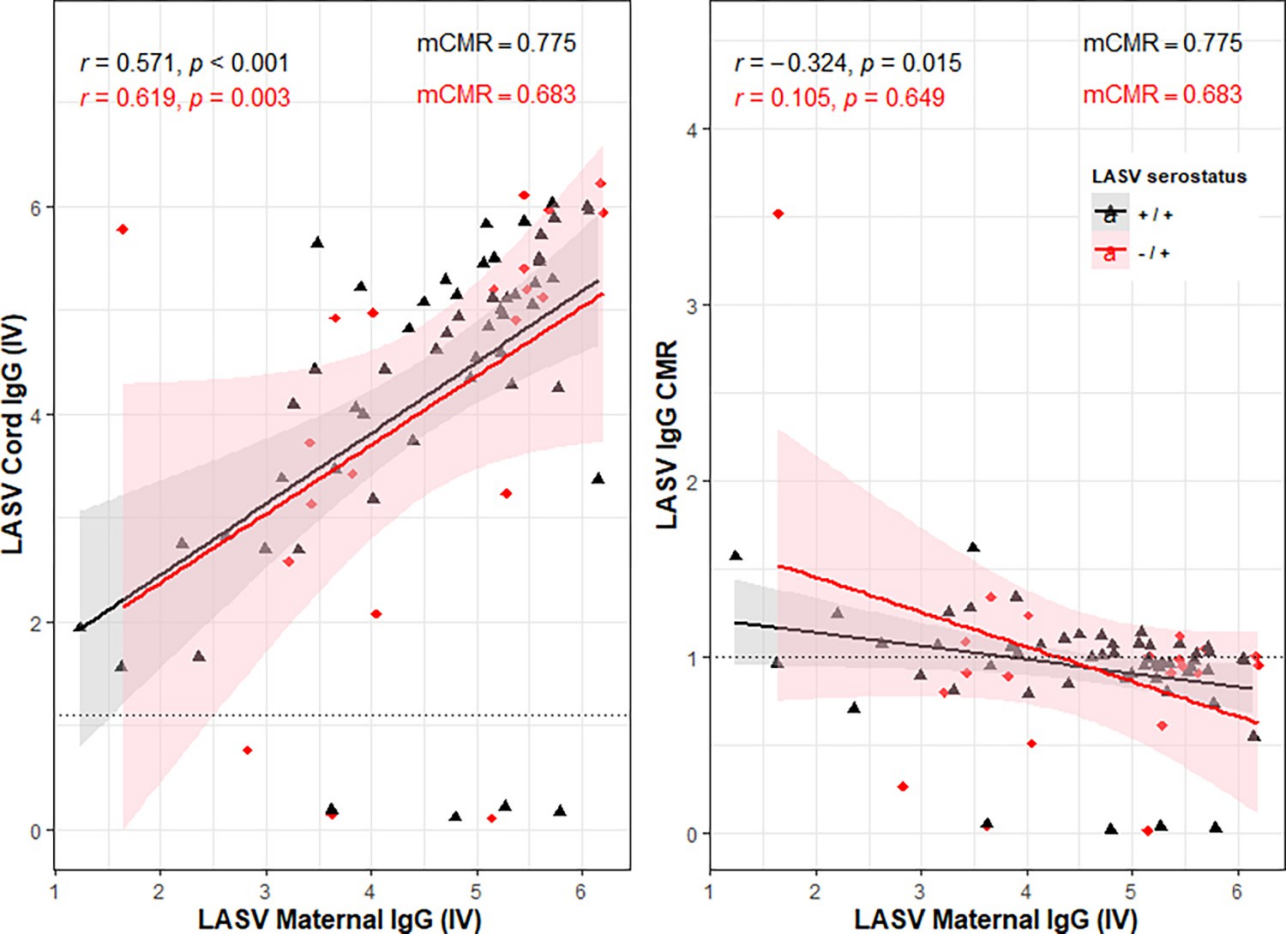
Correlation plots for LASV IgG assessed by Spearman’s correlation (r) using linear regression. Note: +/+ indicates seropositive from baseline. -/+ indicates seroconversion. The dotted black horizontal line indicates the line of efficient transfer (CMR = 1); IV- index value, an arbitrary antibody concentration unit based on manufacturer’s guide; mCMR- median cord maternal ratio; black and red lines are regression lines with the corresponding 95% confidence interval fitted to the regression line.

Given that our LASV IgG seropositive cohort consisted of women who were seropositive at the start of pregnancy (that is, women with pre-existing LASV IgG antibodies), and women who had seroconverted during pregnancy (that is, women with ‘*de novo’* LASV IgG antibodies); we further explored the relationship between LASV maternal concentrations and cord maternal ratios while accounting for LASV seroconversion. We found that amongst mothers with pre-existing antibodies, transfer efficiency was higher with a median CMR of 0.775 [95% CI: 0.60–0.99] compared to those who seroconverted (0.683 [95% CI: 0.42–1.12]), S2 Table. The correlation between maternal CMR or LASV IgG cord concentrations with maternal LASV IgG concentrations while accounting for seroconversion is depicted in Fig 3. The data is also presented in panels in S1 Fig for easy visualisation.

**Fig 4:**
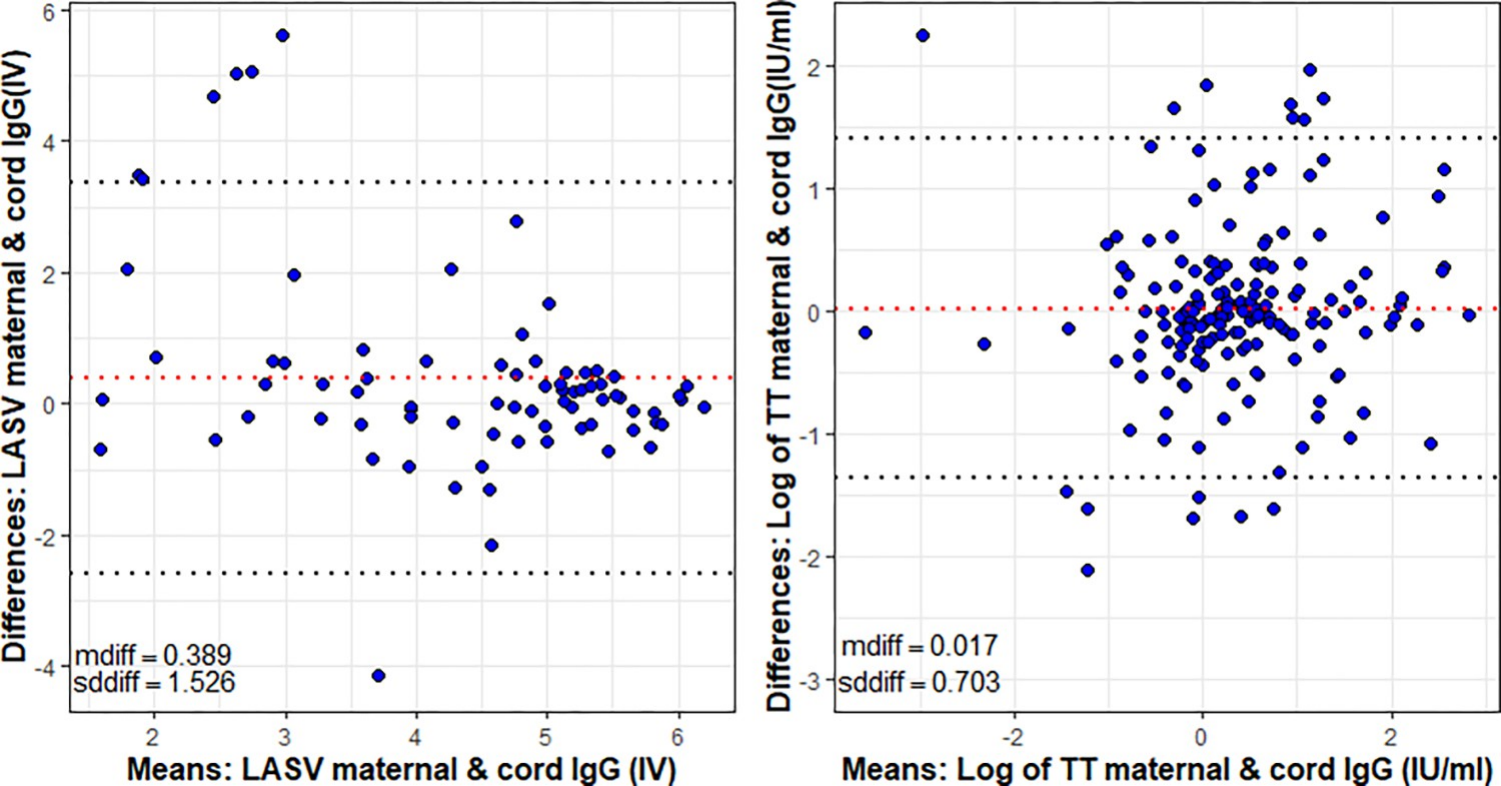
Bland-Altman plot showing agreement between maternal and cord Lassa and TT IgG antibodies. Note: IV- index value; dotted black lines indicate limits of agreement (± 2sddiff); dotted red line indicates mean difference between maternal and cord concentrations; mdiff- mean difference; sddiff- standard deviation of differences.

We used Bland-Altman (B-A) plots to assess the level of agreement between maternal and cord concentrations, Fig 4. For LASV IgG, there is a good level of agreement between maternal and cord levels. The mean difference between maternal and cord levels was 0.389 ± 1.53, with approximately 95% of the data points within the limits of agreement (± 2SD). There was no visual trend, the variability was consistent across the graph, and the difference between maternal and cord concentrations was stable across the graph. The mean difference between maternal and cord TT IgG levels was 0.017 ± 0.703, with approximately 95% of data points within the limits of agreement. However, while there is agreement between maternal and cord concentrations for TT IgG, there was a visual trend with increased variability at higher maternal concentrations, suggesting that at higher maternal concentrations, the difference between maternal and cord concentrations increased.

### Factors associated with changes in LASV IgG transfer ratio (CMR)

We assessed the relationship between LASV IgG transfer ratios and various maternal and neonatal factors. Linear regression was performed and showed that in both the univariate and multivariable models, LASV maternal IgG concentration was the sole factor significantly associated with changes in LASV IgG CMR, Table 2.

**Table 2:** Linear regression analysis of the effect of different factors on the transfer ratio of LASV IgG antibodies.

Factor	N	β crude [95% CI]	P crude	β adjusted [95% CI]	P adjusted^a^
Maternal age (years)	77	-0·005 [-0·02 to 0·01]	0·582	—	—
Parity	77	-0·001[-0·13 to 0·13]	0·983	—	—
Maternal LASV IgG conc	77	-0·112 [-0·19 to -0·02]	0·009	-0·111 [-0·19 to -0·03]	0·011
Fever during pregnancy					
No	60	Baseline		—	—
Yes	17	-0·018 [-0·26 to 0·23]	0·881	—	—
Positive history of LF					
No	75	Baseline		—	—
Yes	2	0·012 [-0·62 to 0·65]	0·969	—	—
Malaria during pregnancy					
No	43	Baseline		—	—
Yes	34	0·002 [-0·21 to 0·21]	0·983	—	—
Hypertension					
No	74	Baseline		Baseline	
Yes	3	-0·401 [-0·91 to 0·12]	0·126	-0·397 [-0·89 to 0·09]	0·116
Pregnancy-induced hypertension					
No	74	Baseline		—	—
Yes	3	0·074 [-0·45 to 0·59]	0·780	—	—
Diabetes mellitus					
No	75	Baseline		—	—
Yes	2	0·156 [-0·48 to 0·79]	0·627	—	—
HIV–infected					
No	70	Baseline		—	—
Yes	7	0·203 [-0·15 to 0·55]	0·251	—	—
Gestational age^b^ (weeks)					
≥ 37	65	Baseline		—	—
< 37	6	0·036 [-0·39 to 0·47]	0·868	—	—
Birthweight (Kg)					
≥ 2·5	71	Baseline		—	—
< 2·5	6	0·017 [-0·39 to 0·43]	0·937	—	—
Sex at birth^c^					
Male	34	Baseline		—	—
Female	42	0·081 [-0·12 to 0·29]	0·444	—	—

Note: β- regression coefficient showing change in LASV IgG CMR; CI- confidence interval; CMR- cord–maternal ratio; conc- concentration; IgG- immunoglobulin G; LASV- Lassa virus; LF- Lassa fever; N- total number of pregnant women included in analysis.

^a^ Adjusted for factors which were marginally significant in the univariate analysis (p<0.25).

^b^ Based on Ballard’s score, data missing for 6 women.

^c^ Data missing for 1 woman.

We found that for every point increase in maternal LASV IgG concentration, there was a 0.111 decrease (95% CI: -0·194 to -0·027, p = 0.011) in cord maternal ratio. Maternal conditions such as HIV infection, malaria, pregestational diabetes, chronic hypertension, gestational diabetes, or hypertensive disorders of pregnancy were not associated with changes in LASV IgG CMR. Similarly, neonatal conditions such as prematurity and low birth weight were not significantly associated with changes in LASV IgG CMR. When accounting for maternal LASV IgG serostatus, we found that amongst women with pre-existing antibodies increasing maternal LASV IgG concentrations were associated with a marginally significant decrease in cord maternal ratio (-0.072 [95% CI: -0.14 to 0.001], p = 0.052), S4A Table; while amongst women who seroconverted none of the factors was significantly associated with changes in cord maternal ratio, S4B Table.

### Factors associated with changes in cord LASV IgG levels

We evaluated the relationship between clinical variables and cord IgG concentrations, Table 3. We found that increasing levels of maternal LASV IgG concentration were associated with a significant increase in cord concentrations (p = 0.001); cord concentrations increased by a unit of 0.676 [95% CI: 0.370 to 0.980] for every point increase in maternal concentration.

**Table 3:** Linear regression analysis of the effect of maternal or neonatal characteristics on cord LASV IgG concentrations.

Factor	N	β crude [95% CI]	P crude	β adjusted^a^ [95% CI]	P adjusted^a^
Maternal age (years)	77	-0·024 [-0·09 to 0·05]	0·521	—	—
Parity	77	-0·093 [-0·29 to 0·10]	0·346	—	—
Maternal LASV IgG conc	77	0·681 [0·39 to 0·98]	<0·001	0·676 [0·37 to 0·98]	0·001
Fever during pregnancy					
No	60	Baseline		—	—
Yes	17	0·336 [-0·60 to 1·27]	0·477	—	—
Positive history of LF					
No	75	Baseline		—	—
Yes	2	1·253 [-1·17 to 3·68]	0·307	—	—
Malaria during pregnancy					
No	43	Baseline		—	—
Yes	34	-0·053 [-0·85 to 0·74]	0·895	—	—
Hypertension					
No	74	Baseline		Baseline	
Yes	3	-1·778 [-3·74 to 0·19]	0·076	-1·810 [-3·57 to -0·05]	0·044
Pregnancy-induced hypertension					
No	74	Baseline		—	—
Yes	3	0·826 [-1·17 to 2·83]	0·413	—	—
Diabetes mellitus					
No	75	Baseline		—	—
Yes	2	-0·115 [-2·56 to 2·33]	0·926	—	—
HIV–infected					
No	70	Baseline		—	—
Yes	7	0·644 [-0·70 to 1·99]	0·343	—	—
Gestational age^b^ (weeks)					
≥ 37	65	Baseline		—	—
< 37	6	-0·938 [-2·53 to 0·66]	0·279	—	—
Birthweight (Kg)					
≥ 2·5	71	Baseline		Baseline	
< 2·5	6	-0·862 [-2·44 to 0·71]	0·246	-0·096 [-1·53 to 1·34]	0·893
Sex at birth^c^					
Male	34	Baseline		—	—
Female	42	-0·334 [-1·13 to 0·46]	0·407	—	—

Note: β- regression coefficient showing change in cord LASV IgG concentration; CI- confidence interval; conc- concentration; IgG- immunoglobulin G; LASV- Lassa virus; LF- Lassa fever; N- total number of pregnant women included in analysis.

^a^ Adjusted for factors marginally significant in the univariate regression (crude p<0·25), except collinear terms.

^b^ Based on Ballard’s’ score, data missing for 6 women.

^c^ Data missing for 1 woman.

Chronic hypertension was also associated with a decrease in cord concentration, (-1.810 [95% CI: -3.57 to -0·05], p = 0.044). Other maternal and neonatal conditions were not found to be associated with significant changes in LASV IgG cord concentrations. When accounting for maternal LASV IgG serostatus, we found that amongst women with pre-existing antibodies increasing maternal LASV IgG concentrations was associated with a significant increase in LASV IgG cord concentrations (0.705 [95% CI: 0.31 to 1.10], p = 0.001), S5A Table; while amongst women who seroconverted none of the variables were significantly associated with changes in cord concentrations, S5B Table.

Amongst the 77 seropositive women with singleton deliveries, seven neonates were LASV IgG seronegative. These seven mother-child pairs also had a LASV cord-maternal ratio below one, with a mean of 0.068 [range: 0.021–0.269] and a median of 0.04, which was significantly lower than the overall median CMR of 0.753 [95% CI: 0.600–0.940]. We evaluated for possible factors that may be associated with LASV IgG cord seronegativity (index value of ≤0.9 in cord samples). Using Fisher’s exact test, we did not identify any factors which were significantly associated with cord LASV seronegativity, Table 4.

**Table 4:** Factors associated with cord LASV IgG seronegativity (cord index value ≤ 0.9).

Characteristics	N	LASV IgG seronegative cord samples (%)	LASV IgG seropositive cord samples (%)	P value^a^
Total	75	7	68	
Maternal age (years), [range]		32·3 ± 6·4 [20–41]	31·6 ± 5·3 [20–41]	0·785
Lives in a rural area				
No	37	4 (57·1)	33 (48·5)	
Yes	38	3 (42·9)	35 (51·5)	0·711
Education				
Post–secondary	49	3 (42·9)	46 (67·7)	
No post–secondary	26	4 (57·1)	22 (32·4)	0·227
Parity				
Primigravida	16	0	16 (23·5)	
Multigravida	59	7 (100)	52 (76·5)	0·334
Fever during pregnancy				
No	58	6 (85·7)	52 (76·5)	
Yes	17	1 (14·3)	16 (23·5)	1
Malaria during pregnancy				
No	43	5 (71·4)	38 (55·9)	
Yes	32	2 (28·6)	30 (44·1)	0·692
Hypertension				
No	69	6 (85·7)	63 (92·7)	
Yes	6	1 (14·3)	5 (7·4)	0·456
Diabetes mellitus				
No	73	7 (100)	66 (97·1)	
Yes	2	0	2 (2·9)	1
HIV–infected				
No	68	7 (100)	61 (89·7)	
Yes	7	0	7 (10·3)	1
Gestational age^b^ (weeks)				
≥ 37	64	7 (100)	57 (91·9)	
< 37	5	0	5 (8·1)	1
Birthweight^c^ (kg)				
≥ 2·5	69	7 (100)	62 (92·5)	
< 2·5	5	0	5 (7·5)	1
Sex at birth^d^				
Male	33	3 (42·9)	30 (44·8)	
Female	41	4 (57·1)	37 (55·2)	1
Maternal serostatus				
Positive unchanged	53	4 (57·1)	49 (72·1)	
Seroconverted	22	3 (42·9)	19 (27·9)	0·412
TT CMR below 1				
No	34	4 (57·1)	30 (44·1)	
Yes	41	3 (42·9)	38 (55·9)	0·695

Note: Data presented as n (%) IgG, where n is the number of cord samples which were seropositive or seronegative. CMR- cord-maternal ratio; IgG- immunoglobulin G; LASV- Lassa virus; N- total number of LASV IgG seropositive pregnant women included in analysis; Post-sec indicates post–secondary education; TT- tetanus toxoid.

^a^ Fisher’s exact P value and Student’s t test as appropriate.

^b^ Based on Ballard’s score, data missing for 6 women.

^c^ Data missing for 3 women.

We did not perform a regression analysis because most of the factors had either 100% or 0% proportions and the sample size for seronegativity was less than 10. We reviewed the transfer ratios and cord concentrations for TT IgG in these seven neonates and found that for all seven neonates, the TT cord concentrations were within protective ranges, that is, they were all TT IgG seropositive [range: 0.79–7.58].

We additionally evaluated factors associated with transfer in mothers who were LASV seronegative and we found that similar to the LASV seropositive population, there was a significant positive correlation between maternal and cord samples (r = 0.865, p<0.001) and a negative correlation between transfer ratio and maternal concentrations (r = -0.036, p = 0.708). However, we did not find any factors significantly associated with changes in transfer efficiency or cord concentrations S4C and S5C Tables.

### Factors associated with changes in TT IgG placental transfer and cord antibody levels

Ninety-eight per cent (98%) of neonates were TT IgG seropositive, this means 98% of neonates had protective levels for TT IgG. Given that most neonates and mothers in our cohort had protective levels of TT IgG we performed a logistic regression to evaluate factors which influence changes in TT cord antibody levels. A multivariable linear regression model suggested that cord TT IgG concentrations increased by 0.669 (95% CI: 0.570 to 0.770, p<0.001) for every unit increase in maternal TT IgG concentrations, Table 5. Whereas cord TT IgG concentrations decreased by 1.7% (95% CI: 0.4% to 4.0%, p = 0.033) with increasing maternal age, and mothers with less than two TT vaccinations had a decrease of 39.4% (95% CI: 9.0% to 69.0%, p = 0.010) in TT IgG cord concentrations compared to those who had received two or more TT vaccinations. However, when accounting for multiple comparisons using the Bonferroni correction, only maternal TT IgG concentration was significantly correlated with changes in cord TT IgG concentrations.

**Table 5:** Linear regression analysis for the effect of maternal or neonatal characteristics on TT IgG cord concentrations.

Factor	N	β crude [95% CI]	P crude	β adjusted [95% CI]	P adjusted^a^
Maternal age (years) [range]	170	-0·041 [-0·07 to 0·01]	0·009	-0·017 [-0·04 to 0·004]	0·033
Parity^b^	170	0·135 [-0·34 to 0·07]	0·189	—	—
Maternal LASV IgG conc	170	-0·018 [-0·32 to 0·28]	0·845	—	—
Maternal TT IgG conc	170	0·710 [0·62 to 0·80]	<0·001	0·669 [0·57 to 0·77]	<0·001
Previous history of LF					
No	166	Baseline		—	—
Yes	4	-0·346 [-1·35 to 0·63]	0·487	—	—
Fever during pregnancy					
No	136	Baseline		—	—
Yes	34	0·08 [-0·29 to 0·45]	0·684	—	—
Malaria during pregnancy					
No	104	Baseline		—	—
Yes	66	0·045 [-0·26 to 0·35]	0·771	—	—
Hypertension					
No	160	Baseline		—	—
Yes	10	-0·178 [-1·05 to 0·70]	0·689	—	—
Pregnancy-induced hypertension					
No	166	Baseline		Baseline	
Yes	4	0·953 [-0·02 to 1·92]	0·054	0·210 [-0·43 to 0·85]	0·520
Diabetes mellitus					
No	164	Baseline		—	—
Yes	6	0·464 [-0·51 to 1·44]	0·350	—	—
Gestational diabetes					
No	168	Baseline		—	—
Yes	2	0·221 [-1·15 to1·59]	0·752	—	—
HIV–infected					
No	162	Baseline		—	—
Yes	8	0·254 [-0·45 to 0·96]	0·474	—	—
Total TT received					
≥ 2	148	Baseline		Baseline	
< 2	22	-0·675 [-1·11 to -0·24]	0·003	-0·394 [-0·69 to -0·09]	0·010
Gestational age (weeks)^c^					
≥ 37	139	Baseline		—	—
< 37	13	-0·067 [-0·59 to 0·47]	0·804	—	—
Birthweight (Kg)^d^					
≥ 2·5	158	Baseline		Baseline	
< 2·5	11	-0·451 [-1·05 to 0·15]	0·141	-0·006 [-0·40 to 0·39]	0·977
Sex at birth^d^					
Male	82	Baseline		Baseline	
Female	87	-0·205 [-0·50 to 0·09]	0·179	-0·056 [-0·25 to 0·14]	0·580

Note: For statistical significance, **Bonferroni p<0.008.** β—regression coefficient showing change in TT IgG cord concentrations; CI- confidence interval; conc- concentration; IgG- immunoglobulin G; LASV- Lassa virus; LF- Lassa fever; N- total number of pregnant women included in analysis.

^a^ Adjusted for factors marginally significant in the univariate regression (crude p<0·25).

^b^ Age and parity are collinear, so parity excluded from multivariable analysis.

^c^ Based on Ballard’s score, data missing for 18 women.

^d^ Data missing for 1 woman.

## Discussion

In this cohort, LASV IgG was transferred at 75.3% compared to 99.1% for TT IgG. Cord LASV IgG was positively correlated with maternal IgG levels with a good level of agreement between maternal and cord concentrations. Similar to reports on other antigens [38,39], both LASV and TT show a positive correlation between maternal and cord antibodies and a negative correlation between maternal IgG levels and transfer ratios; although the effect was more significant for TT IgG than for LASV IgG. IgG antibodies are differentially transported across the placental barrier, with some antibodies more efficiently transferred than others. For instance, studies report that measles antibodies are more efficiently transferred than HIV [40], poliovirus [40], or coxsackievirus antibodies [40]. While the mechanisms are unclear, antibody specific factors such as IgG subclass differences and affinities, and infection-related antibody glycosylation have been suggested as potential reasons for variable antibody transfer [11,41–43]. This differential transfer may explain why TT IgG antibodies were more efficiently transported (CMR 0.991) than LASV IgG antibodies (CMR 0.753) and requires further enquiry.

We found 45.7% of mothers were seropositive at delivery and this high prevalence can be explained by the fact that the study was conducted in a region known to have high annual incidences of Lassa fever [16]. In this region, the reported prevalence of LASV IgG in the general population from cross-sectional studies was 58.2% [44].

Our seropositive population consisted of mothers who had pre-existing antibodies and mothers with ‘de novo’ antibodies (seroconverters). While transfer ratios in both seroconverters and those with pre-existing antibodies were negatively correlated with maternal concentrations; the transfer ratio was lower amongst seroconverters (0.683 [95% CI: 0.42–1.12]), compared to those with pre-existing antibodies (0.775 [95% CI: 0.60–0.99]), suggesting that transfer efficiency was lower in seroconverters. Furthermore, the wider confidence interval amongst seroconverters suggests there may be more variability in transfer amongst mothers with ‘de novo’ antibodies. A possible explanation for this is that, in women who seroconvert, there is a switch in antibody subclass production such that, for these mothers, different IgG isotypes are dominant at different times depending on the time at which seroconversion occurred. Transfer efficiency is affected by the IgG subclasses [45,46] and antibody affinity [47]; so depending on the time of seroconversion, one or other subclass may dominate. An understanding of the differences in antibody subclass production and kinetics for LASV IgG in both seroconverting mothers and mothers who were seropositive before pregnancy may help explain the differences observed. Additionally, given that, there were few seroconverters in our study, further investigation with a larger cohort is needed to provide a more accurate picture of the effect of acute or recent Lassa infection.

Maternal conditions or infections such as human immunodeficiency virus (HIV) infection and placental malaria have been known to decrease antibody transfer efficiency [24,26,27]. In this cohort, HIV infection was not associated with depressed LASV IgG or TT IgG transfer. A possible reason for this is that approximately 80% of the HIV-infected mothers were on antiretroviral therapy (ART), and studies have reported normalisation of humoral responses with a decrease in hypergammaglobulinemia with the use of ART [48–50]. Similarly, both TT and LASV IgG transfer were unaffected by malaria infection in our cohort. We did not evaluate for placental malaria, but studies on peripheral malaria suggest that even peripheral malaria may result in decreased transfer efficiency [26]. TT IgG may have been unaffected because over 90% of the malaria-exposed pregnant women had received at least two doses of TT vaccination and 91% of these had received malaria treatment with either quinine or artemisinin combination therapy. It is possible that LASV IgG transfer was unaffected by malaria because all participants in that subgroup had received antimalarials (quinine). Further studies are, however, needed to understand the effect of malaria on LASV IgG transfer.

Maternal conditions such as chronic hypertension, pregnancy-induced hypertension, pregestational and gestational diabetes were not significantly associated with changes in transfer efficiency of TT IgG. However, chronic hypertension was marginally associated with a significant decrease in cord LASV IgG concentrations. So far, evidence on the effect of diabetes and hypertension are conflicting with some studies reporting improved antibody transfer efficiency in both these conditions [38], whereas others suggest that both hypertension and diabetes cause a decrease in antibody transfer [51]. Given that, within our cohort, there were relatively few hypertensive and diabetic women, an avenue for further research would be to evaluate in a larger cohort the effects of hypertension and diabetes on the transfer of LASV IgG antibodies, and vaccine antigens such as tetanus toxoid.

### Limitations

Some limitations to consider include the fact that we did not assess the neutralising capacity of the antibodies transferred in this cohort, and cannot provide evidence on the protective nature of the antibodies transferred to new-borns. An IgG neutralisation test would facilitate assessment of the neutralising capacity of the transferred antibodies. However, the study addresses the question on the efficiency of transfer of LASV IgG antibodies, providing evidence that can be used for antibody-based vaccine design. Further studies are however needed, to understand if the antibodies transferred were neutralising and the decay of these antibodies in new-borns and infants; to provide a better understanding of the time for vaccination for both mothers and their new-borns.

An added limitation is that we did not evaluate for IgG antibody subclasses and LASV antigen specificity which would improve our understanding of their effect on transfer efficiency. Further studies are needed to evaluate antibody subclasses as well as explore antigen specificity to better characterise the antibodies transferred and evaluate the rate of transfer for each subclass.

Our cohort had few seroconverts, as such further investigation with a larger cohort is needed to provide a more accurate picture of the effect of acute or recent Lassa infection and provide evidence on the stability of transfer between *‘de novo’* and pre-existing antibodies. Additionally, because our seroconverting population was identified based on two study time points, the exact time at which seroconversion occurred is unknown. Future studies designed to estimate time at which seroconversion occurred and evaluate the effect on transfer efficiency would be beneficial.

Our study was unable to identify any factors associated with cord LASV IgG seronegativity but given that the sample size for cord seronegativity was small (<10 neonates), further studies are needed to identify factors that may be associated with cord seronegativity.

## Conclusion

This study demonstrates a transplacental transfer of LASV IgG of 75.3% and shows that maternal antibody levels play an important role in determining efficiency of transfer. The variation between transplacental transfer of pre-existing antibodies and ‘*de novo’* antibodies observed here suggest that transfer efficiency may be less stable in acute or recent infection, as such timing of vaccination before pregnancy that is in women of childbearing age may be more appropriate than vaccination during pregnancy. This evidence is preliminary, but we believe is an important first step to understanding the kinetics of Lassa antibodies.

## Supporting information

S1 Table Baseline characteristics of mothers and neonates by maternal LASV IgG serostatus. (DOCX)

S2 Table LASV IgG concentrations and cord maternal ratio by various maternal and neonate characteristics. (DOCX)

S3 Table TT IgG concentrations and cord maternal ratio by various maternal and neonate characteristics. (DOCX)

S4 Table **(A) Linear regression analysis of the effect of different factors on the transfer ratio of LASV IgG antibodies amongst mothers with pre-existing antibodies**. Note: β- regression coefficient showing change in cord LASV IgG concentration; CI- confidence interval; CMR- cord–maternal ratio; conc- concentration; IgG- immunoglobulin G; LASV- Lassa virus; LF- Lassa fever; N- total number of pregnant women included in analysis. a Adjusted for factors marginally significant in the univariate regression (crude p<0·25), except collinear terms. **(B) Linear regression analysis of the effect of different factors on the transfer ratio of LASV IgG antibodies amongst mothers who seroconverted.** Note: β- regression coefficient showing change in cord LASV IgG concentration; CI- confidence interval; CMR- cord–maternal ratio; conc- concentration; IgG- immunoglobulin G; LASV- Lassa virus; LF- Lassa fever; N- total number of pregnant women included in analysis. a Adjusted for factors marginally significant in the univariate regression (crude p<0·25), except collinear terms. ND–Multivariable regression not performed as not of the variables satisfied the criteria for inclusion in a multivariable model. No factor or variable was significantly associated with changes in CMR for Seroconverters. **(C) Linear regression analysis of the effect of different factors on the transfer ratio of LASV IgG antibodies amongst mothers who were seronegative.** Note: β- regression coefficient showing change in cord LASV IgG concentration; CI- confidence interval; CMR- cord–maternal ratio; conc- concentration; IgG- immunoglobulin G; LASV- Lassa virus; LF- Lassa fever; N- total number of pregnant women included in analysis. a Adjusted for factors marginally significant in the univariate regression (crude p<0·25), except collinear terms. ND–Multivariable regression not performed as not of the variables satisfied the criteria for inclusion in a multivariable model. No factor or variable was significantly associated with changes in CMR for Seronegative mothers.
(DOCX)

S5 Table **(A) Linear regression analysis of the effect of different factors cord LASV IgG concentrations amongst mothers with pre-existing antibodies**. Note: β- regression coefficient showing change in cord LASV IgG concentration; CI- confidence interval; conc- concentration; IgG- immunoglobulin G; LASV- Lassa virus; LF- Lassa fever; N- total number of pregnant women included in analysis. a Adjusted for factors marginally significant in the univariate regression (crude p<0·25), except collinear terms. **(B) Linear regression analysis of the effect of different factors on cord LASV IgG concentrations amongst mothers who seroconverted**. Note: β- regression coefficient showing change in cord LASV IgG concentration; CI- confidence interval; conc- concentration; IgG- immunoglobulin G; LASV- Lassa virus; LF- Lassa fever; N- total number of pregnant women included in analysis. a Adjusted for factors marginally significant in the univariate regression (crude p<0·25), except collinear terms. ND–Multivariable regression not performed as not of the variables satisfied the criteria for inclusion in a multivariable model. No factor or variable was significantly associated with changes in cord IgG concentrations for Seroconverters. **(C) Linear regression analysis of the effect of different factors on cord LASV IgG concentrations amongst mothers who were seronegative**. Note: β- regression coefficient showing change in cord LASV IgG concentration; CI- confidence interval; conc- concentration; IgG- immunoglobulin G; LASV- Lassa virus; LF- Lassa fever; N- total number of pregnant women included in analysis. a Adjusted for factors marginally significant in the univariate regression (crude p<0·25), except collinear terms. ND–Multivariable regression not performed as not of the variables satisfied the criteria for inclusion in a multivariable model. No factor or variable was significantly associated with changes in cord IgG concentrations for Seronegative mothers.
(DOCX)

S1 Fig Correlation plots for LASV IgG assessed by Spearman’s correlation (r) using linear regression. Note: The figure evaluates the relationship between maternal LASV IgG concentration and placental transfer measured as the CMR and the relationship between maternal LASV IgG with cord LASV IgG concentration while accounting for seroconversion. Black indicates seropositive from baseline and red indicates seroconversion. The dotted black horizontal line indicates the line of efficient transfer (CMR = 1); IV- index value, an arbitrary antibody concentration unit based on manufacturer’s guide; mCMR- median cord maternal ratio; black and red lines are regression lines with the corresponding 95% confidence interval fitted to the regression line.
(TIF)
