# Supplementary material for: Transplacental transfer of Lassa IgG antibodies in pregnant women in Southern Nigeria: A prospective hospital-based cohort study
Source: PLoS Negl Trop Dis. 2023 Apr 13;17(4):e0011209. doi: 10.1371/journal.pntd.0011209 (PMC10129015; doi:10.1371/journal.pntd.0011209)
Supplement: S1 Table — (DOCX) [file pntd.0011209.s001.docx]

| Characteristic | Seropositive from baseline  n (%) | Seroconverters  n (%) | Seronegative  n (%) | Total  N (%) |
| --- | --- | --- | --- | --- |
| N | 55 | 22 | 93 | 170 |
| Maternal age (years) |  |  |  |  |
| Mean ± SD [range] | 31.3±5.12 [20-41] | 32.7±5.9 [21 -41] | 31.5± 4.4 [22 -46] | 31·6 ± 4·8 [20 – 46] |
| ≤ 25 | 9 (16.4) | 2 (9.1) | 10(10.8) | 21 (12·4) |
| 26 - 30 | 14 (25.5) | 8(36.4) | 29(31.2) | 51 (30·0) |
| 31 - 35 | 26 (47.3) | 4(18.2) | 42(45.2) | 72 (42·4) |
| > 36 | 6 (10.8) | 8(36.4) | 12(12.8) | 26 (15·3) |
| Lives in rural area | 28 (50.9) | 10(45.5) | 56(60.2) | 94 (55·3) |
| Education |  |  |  |  |
| Primary | 3 (5.5) | 3(13.6) | 2(2.2) | 8 (4·7) |
| Secondary | 15 (27.3) | 5(22.7) | 13(14.0) | 33 (19·4) |
| Post - secondary | 37 (67.3) | 14(63.6) | 78(83.9) | 129(75·8) |
| Parity |  |  |  |  |
| 0 | 12 (21.8) | 5(22.7) | 19(20.4) | 35(20·6) |
| 1 – 2 | 22 (40.0) | 7(31.8) | 42(45.2) | 72(42·4) |
| ≥ 3 | 21 (38.2) | 10(45.5) | 32(34.4) | 63 (37·0) |
| Gestational age at baseline (weeks) |  |  |  |  |
| Median (IQR) | 26 [16-36] | 18 [10 - 27] | 26 [16 -37] | 24 [18 – 31] |
| ≤ 13 | 6 (11.5) | 4(18.2) | 10(11.1) | 20 (12.3) |
| 14 – 27 | 25 (48.1) | 15(68.2) | 46(51.1) | 85 (51.8) |
| >28 | 21 (40.4) | 3(13.6) | 34(37.8) | 59 (35.9) |
| Positive history of fever during pregnancy | 11 (20.0) | 6(27.3) | 17(18.3) | 34 (20·0) |
| Positive history of prior LF | 1 (1.8) | 1(4.5) | 2(2.2) | 4 (2·4) |
| Positive history of malaria during pregnancy | 25 (45.5) | 9(40.9) | 34 (36.6) | 67 (30·4) |
| HIV - infected | 6 (10.9) | 1(4.5) | 1(1.1) | 8 (4·7) |
| Diabetes Mellitus | 2 (3.6) | 0 | 2(2.2) | 4 (2·4) |
| Gestational diabetes | 0 | 0 | 2(2.2) | 2 (1·2) |
| Hypertension | 2 (3.6) | 1(4.5) | 2(2.2) | 5 (2·9) |
| Pregnancy-induced hypertension | 1 (1.8) | 2(9.1) | 1(1.1) | 4 (2·4) |
| Total number of TT vaccinations received |  |  |  |  |
| ≥ 2 | 44 (79.9) | 20(90.9) | 84(90.3) | 148 (87·1) |
| < 2 | 11 (20.1) | 2(9.1) | 9 (9.7) | 22 (12·9) |
| Maternal LASV IgG GMC [95% CI] | 4·40 [3·72 - 5·01] | 4·32 [4·04 - 4·79] | 0.161 [0.14 -0.18] | — |
| Maternal TT IgG GMT [95% CI] | 1·44 [1·07 - 1·93] | 1·87 [1·15 - 3·02] | 1·34 [1·08 - 1·66] | 1·46 [1·25 - 1·71] |
| New-born characteristics |  |  |  |  |
| Gestational age at birth (weeks) | |  |  |  |
| Mean ± SD [range] | 39.4 ± 2.0 [34-43] | 39.3 ± 2.03 [35 -42] | 39.1± 2.0 [29 -41] | 39·2 ± 2·0 [29 – 43] |
| ≥ 37 | 45 (93.8) | 20(90.9) | 73 (91.2) | 139 (91·4) |
| < 37 | 3 (6.2) | 2(9.1) | 7 (8.7) | 13 (8·6) |
| Birthweight (Kg) |  |  |  |  |
| Mean ± SD [range] | 3.2 ± 0.4 [2.1 -4.2] | 3.2 ± 0.4 [2.4 -4.0] | 3.2 ± 0.5 [0.9 -4.7] | 3·2 ± 0·5 [0·9 – 4·7] |
| ≥ 2·5 | 50 (92.6) | 21(95.5) | 87 (93.5) | 158 (93·5) |
| < 2·5 | 4 (7.4) | 1(4.5) | 6 (6.5) | 11 (6·5) |
| LASV IgG median CMR [95% CI] | 0·775 [0·60 - 0·99] | 0·683 [0·42 - 1·12] | 0.747 [0.60 -0.95] | — |
| Cord LASV IgG GMC [95% CI] | 3·42 [2·68 - 4·36] | 2·92 [1·72 - 4·93] | 0.146 [0.13 -0.17] | — |
| Cord TT IgG GMT [95% CI] | 1·34 [1·00 - 1·79] | 1·73 [1·14 - 2·63] | 1·41 [1·15 - 1·73] | 1·43 [1·24 - 1·67] |
| TT IgG median CMR | 0·92 [0·75 - 1·13] | 0·91 [0·71 - 1·18] | 1·06 [0·89 - 1·26] | 0·991 [0·88 - 1·11] |
