# Supplementary material for: Transplacental transfer of Lassa IgG antibodies in pregnant women in Southern Nigeria: A prospective hospital-based cohort study
Source: PLoS Negl Trop Dis. 2023 Apr 13;17(4):e0011209. doi: 10.1371/journal.pntd.0011209 (PMC10129015; doi:10.1371/journal.pntd.0011209)
Supplement: S2 Table — (DOCX) [file pntd.0011209.s002.docx]

| Characteristics | LASV seropositive | Maternal GMC  [95% CI] | Cord Maternal Ratio [95% CI] | Cord GMC  [95% CI] |
| --- | --- | --- | --- | --- |
| **Total** | 77 | 4·352 [4·04 - 4·68] | 0·753 [0·60 - 0·94] | 3·275 [2·62 - 4·09] |
| **Maternal age (years)** |  |  |  |  |
| ≤ 30 | 33 | 4·33 [3·84 - 4·88] | 0·89 [0·73 - 1·10] | 3·84 [3·00 - 4·92] |
| >30 | 44 | 4·41 [4·02 - 4·84] | 0·66 [0·47 - 0·94] | 2·91 [2·06 - 4·11] |
| **Lives in a rural area** |  |  |  |  |
| No | 39 | 4·49 [4·05 - 4·98] | 0·71 [0·49 - 1·02] | 3·14 [2·20 - 4·49] |
| Yes | 38 | 4·26 [3·84 - 4·73] | 0·80 [0·62 - 1·04] | 3·41 [2·57 - 4·52] |
| **Educational level** |  |  |  |  |
| Post - sec | 51 | 4·25 [3·87 - 4·68] | 0·83 [0·67 - 1·03] | 3·49 [2·79 - 4·36] |
| No post - sec | 26 | 4·61 [4·13 - 5·15] | 0·64 [0·39 - 1·04] | 2·93 [1·80 - 4·78] |
| **Occupation** |  |  |  |  |
| Student | 5 | 4·39 [3·01 - 6·39] | 0·41 [0·08 - 2·02] | 1·81 [0·28 - 11·8] |
| Housewife | 13 | 4·13 [3·35 - 5·09] | 0·76 [0·42 - 1·39] | 3·08 [1·61 - 5·90] |
| Health Professional | 7 | 4·57 [3·31 - 6·32] | 0·88 [0·72 - 1·07] | 4·02 [2·64 - 6·13] |
| Informal Sector | 31 | 4·71 [4·34 - 5·11] | 0·75 [0·50 - 1·11] | 3·52 [2·39 - 5·19] |
| Formal Sector | 21 | 3·98 [3·30 - 4·80] | 0·83 [0·57 - 1·21] | 3·26 [2·33 - 4·55] |
| **Parity** |  |  |  |  |
| Primigravida | 17 | 4·70 [4·24 - 5·22] | 0·91 [0·83 - 1·01] | 4·25 [3·75 - 4·82] |
| Multigravida | 60 | 4·29 [3·93 - 4·68] | 0·72 [0·54 - 0·94] | 3·06 [2·32 - 4·03] |
| **Fever during pregnancy** |  |  |  |  |
| No | 60 | 4·30 [3·94 - 4·69] | 0·75 [0·58 - 0·96] | 3·18 [2·47 - 4·11] |
| Yes | 17 | 4·66 [4·18 - 5·20] | 0·78 [0·48 - 1·27] | 3·62 [2·22 - 5·91] |
| **Malaria during pregnancy** | |  |  |  |
| No | 43 | 4·50 [4·15 - 4·89] | 0·69 [0·49 - 0·97] | 3·12 [2·23 - 4·36] |
| Yes | 34 | 4·22 [3·71 - 4·80] | 0·84 [0·65 - 1·09] | 3·50 [2·65 - 4·61] |
| **Hypertension** |  |  |  |  |
| No | 71 | 4·36 [4·03 - 4·71] | 0·77 [0·62 - 0·97] | 3·30 [2·65 - 4·20] |
| Yes | 6 | 4·57 [3·80 - 5·49] | 0·59 [0·19 - 1·80] | 2·70 [0·92 - 7·93] |
| **Diabetes Mellitus** |  | |  |  |
| No | 75 | 4·40 [4·09 - 4·73] | 0·75 [0·59 - 0·93] | 3·26 [2·60 - 4·09] |
| Yes | 2 | 3·51 [2·62 - 4·71] | 1·10 [0·83 - 1·45] | 3·86 [2·81 - 5·28] |
| **HIV - infected** |  |  |  |  |
| No | 70 | 4·42 [4·11 - 4·75] | 0·72 [0·57 - 0·92] | 3·18 [2·50 - 4·05] |
| Yes | 7 | 3·97 [2·65 - 5·95] | 1·11 [0·90 - 1·36] | 4·39 [2·81 - 6·86] |
| **Gestational age at birth** |  |  |  |  |
| ≥ 37 | 65 | 4·52 [4·23 - 4·83] | 0·72 [0·55 - 0·93] | 3·23 [2·50 - 4·19] |
| < 37 | 6 | 3·28 [1·82 - 5·88] | 0·92 [0·62 - 1·38] | 3·02 [1·72 - 5·29] |
| **Birthweight** |  |  |  |  |
| ≥ 2·5 | 71 | 4·48 [4·18 - 4·81] | 0·74 [0·58 - 0·94] | 3·27 [2·57 - 4·17] |
| < 2·5 | 6 | 3·19 [1·95 - 5·21] | 0·96 [0·81 - 1·13] | 3·07 [1·72 - 5·48] |
| **Sex at birth** |  |  |  |  |
| Male | 34 | 4·70 [4·38 - 5·04] | 0·72 [0·51 - 1·03] | 3·39 [2·38 - 4·82] |
| Female | 42 | 4·10 [3·63 - 4·63] | 0·77 [0·57 - 1·04] | 3·15 [2·33 - 4·25] |
| **Maternal LASV serostatus** |  |  |  |  |
| Seropositive at baseline | 55 | 4·40 [3·72 - 5·01] | 0·78 [0·60 - 0·99] | 3·42 [2·68 - 4·36] |
| Seroconverted | 22 | 4·32 [4·04 - 4·79] | 0·68 [0·42 - 1·12] | 2·92 [1·72 - 4·93] |
