# Supplementary material for: Transplacental transfer of Lassa IgG antibodies in pregnant women in Southern Nigeria: A prospective hospital-based cohort study
Source: PLoS Negl Trop Dis. 2023 Apr 13;17(4):e0011209. doi: 10.1371/journal.pntd.0011209 (PMC10129015; doi:10.1371/journal.pntd.0011209)
Supplement: S3 Table — (DOCX) [file pntd.0011209.s003.docx]

| Characteristics | Total | Maternal GMT [95% CI] | Cord Maternal Ratio [95% CI] | Cord GMT  [95% CI] |
| --- | --- | --- | --- | --- |
| **Total** | 170 | 1·464 [1·25 - 1·71] | 0·991 [0·88 - 1·11] | 1·438 [1·24 - 1·67] |
| **Maternal age (years)** |  |  |  |  |
| ≤ 30 | 72 | 1·91 [1·48 - 2·47] | 0·91 [0·77 - 1·07] | 1·78 [1·44 - 2·20] |
| >30 | 98 | 1·16 [0·96 - 1·41] | 1·05 [0·89 - 1·23] | 1·23 [1·01 - 1·51] |
| **Lives in a rural area** |  |  |  |  |
| No | 76 | 1·46 [1·17 - 1·81] | 1·04 [0·86 - 1·25] | 1·56 [1·30 - 1·88] |
| Yes | 94 | 1·42 [1·13 - 1·79] | 0·96 [0·83 - 1·10] | 1·35 [1·08 - 1·68] |
| **Educational level** |  |  |  |  |
| Post - sec | 129 | 1·49 [1·25 - 1·79] | 0·99 [0·87 - 1·14] | 1·50 [1·27 - 1·78] |
| No post - sec | 41 | 1·28 [1·25 - 1·79] | 0·98 [0·77 - 1·24] | 1·27 [0·93 - 1·75] |
| **Occupation** |  |  |  |  |
| Student | 11 | 2·70 [1·28 - 5·70] | 0·94 [0·57 - 1·54] | 2·52 [1·39 - 4·57] |
| Housewife | 28 | 1·21 [0·75 - 1·94] | 0·97 [0·73 - 1·30] | 1·12 [0·64 - 1·94] |
| Health Professional | 20 | 1·55 [1·05 - 2·31] | 0·82 [0·54 - 1·25] | 1·33 [0·83 - 2·12] |
| Informal Sector | 66 | 1·44 [1·13 - 1·84] | 1·02 [0·84 - 1·24] | 1·46 [1·19 - 1·78] |
| Formal Sector | 45 | 1·33 [0·98 - 1·82] | 1·06 [0·86 - 1·29] | 1·49 [1·16 - 1·92] |
| **Parity** |  |  |  |  |
| Primigravida | 35 | 1·86 [1·35 - 2·56] | 0·89 [0·71 - 1·11] | 1·62 [1·08 - 2·43] |
| Multigravida | 135 | 1·34 [1·12 - 1·61] | 1·02 [0·89 - 1·16] | 1·40 [1·19 - 1·63] |
| **Fever during pregnancy** |  |  |  |  |
| No | 136 | 1·38 [1·15 - 1·64] | 1·01 [0·88 - 1·16] | 1·41 [1·21 - 1·65] |
| Yes | 34 | 1·72 [1·21 - 2·45] | 0·92 [0·75 - 1·12] | 1·55 [1·02 - 2·35] |
| **Malaria during pregnancy** | |  |  |  |
| No | 103 | 1·33 [1·09 - 1·62] | 1·07 [0·91 - 1·24] | 1·41 [1·22 - 1·65] |
| Yes | 67 | 1·61 [1·23 - 2·11] | 0·88 [0·75 - 1·04] | 1·48 [1·14 - 1·93] |
| **Hypertension** |  |  |  |  |
| No | 161 | 1·40 [1·18 - 1·65] | 1·01 [0·89 - 1·13] | 1·42 [1·21 - 1·65] |
| Yes | 9 | 2·30 [1·29 - 4·11] | 0·78 [0·56 - 1·09] | 1·85 [0·99 - 3·43] |
| **Diabetes Mellitus** |  |  |  |  |
| No | 164 | 1·42 [1·21 - 1·67] | 0·99 [0·88 - 1·11] | 1·42 [1·22 - 1·65] |
| Yes | 6 | 2·14 [0·81 - 5·64] | 1·02 [0·67 - 1·54] | 2·12 [1·03 - 4·35] |
| **HIV - infected** |  |  |  |  |
| No | 162 | 1·42 [1·21 - 1·67] | 0·99 [0·88 - 1·12] | 1·42 [1·22 - 1·65] |
| Yes | 8 | 1·84 [0·89 - 3·81] | 0·96 [0·64 - 1·45] | 1·85 [0·74 - 4·65] |
| **Gestational age at birth** |  |  |  |  |
| ≥ 37 | 138 | 1·43 [1·22 - 1·68] | 1·00 [0·88 - 1·14] | 1·45 [1·25 - 1·69] |
| < 37 | 14 | 1·48 [0·74 - 2·98] | 0·87 [0·66 - 1·16] | 1·33 [0·66 - 2·69] |
| **Birthweight** |  |  |  |  |
| ≥ 2·5 | 157 | 1·49 [1·26 - 1·76] | 0·98 [0·87 - 1·11] | 1·48 [1·27 - 1·73] |
| < 2·5 | 12 | 0·88 [0·46 - 1·68] | 1·08 [0·62 - 1·89] | 0·95 [0·47 - 1·92] |
| **Sex at birth** |  |  |  |  |
| Male | 82 | 1·56 [1·25 - 1·94] | 0·99 [0·85 - 1·15] | 1·58 [1·26 - 1·97] |
| Female | 87 | 1·33 [1·05 - 1·68] | 0·99 [0·83 - 1·18] | 1·32 [1·08 - 1·60] |
| **LASV maternal IgG conc** |  |  |  |  |
| < 1·1 | 93 | 1·37 [1·12 - 1·68] | 1·06 [0·91 - 1·24] | 1·47 [1·21 - 1·77] |
| ≥ 1·1 | 77 | 1·52 [1·18 - 1·96] | 0·91 [0·77 - 1·08] | 1·41 [1·11 - 1·78] |
| **LASV maternal serostatus** |  |  |  |  |
| Seronegative from baseline | 84 | 1·34 [1·08 - 1·66] | 1·06 [0·89 - 1·26] | 1·41 [1·15 - 1·73] |
| Seroconverted | 22 | 1·87 [1·15 - 3·02] | 0·91 [0·71 - 1·18] | 1·73 [1·14 - 2·63] |
| Seroreverted | 9 | 1·52 [0·62 - 3·69] | 1·07 [0·70 - 1·62] | 1·78 [0·91 - 3·48] |
| Seropositive from baseline | 55 | 1·44 [1·07 - 1·93] | 0·92 [0·75 - 1·13] | 1·34 [1·00 - 1·79] |
| **Total TT received** |  |  |  |  |
| ≥ 2 | 148 | 1·52 [1·30 - 1·78] | 1·01 [0·89 - 1·14] | 1·57 [1·36 - 1·81] |
| < 2 | 22 | 0·97 [0·52 - 1·80] | 0·89 [0·68 - 1·16] | 0·81 [0·44 - 1·48] |
