# Supplementary material for: Transplacental transfer of Lassa IgG antibodies in pregnant women in Southern Nigeria: A prospective hospital-based cohort study
Source: PLoS Negl Trop Dis. 2023 Apr 13;17(4):e0011209. doi: 10.1371/journal.pntd.0011209 (PMC10129015; doi:10.1371/journal.pntd.0011209)
Supplement: S4 Table — (A) Linear regression analysis of the effect of different factors on the transfer ratio of LASV IgG antibodies amongst mothers with pre-existing antibodies. Note: β- regression coefficient showing change in cord LASV IgG concentration; CI- confidence interval; CMR- cord–maternal ratio; conc- concentration; IgG- immunoglobulin G; LASV- Lassa virus; LF- Lassa fever; N- total number of pregnant women included in analysis. a Adjusted for factors marginally significant in the univariate regression (crude p<0·25), except collinear terms. (B) Linear regression analysis of the effect of different factors on the transfer ratio of LASV IgG antibodies amongst mothers who seroconverted. Note: β- regression coefficient showing change in cord LASV IgG concentration; CI- confidence interval; CMR- cord–maternal ratio; conc- concentration; IgG- immunoglobulin G; LASV- Lassa virus; LF- Lassa fever; N- total number of pregnant women included in analysis. a Adjusted for factors marginally significant in the univariate regression (crude p<0·25), except collinear terms. ND–Multivariable regression not performed as not of the variables satisfied the criteria for inclusion in a multivariable model. No factor or variable was significantly associated with changes in CMR for Seroconverters. (C) Linear regression analysis of the effect of different factors on the transfer ratio of LASV IgG antibodies amongst mothers who were seronegative. Note: β- regression coefficient showing change in cord LASV IgG concentration; CI- confidence interval; CMR- cord–maternal ratio; conc- concentration; IgG- immunoglobulin G; LASV- Lassa virus; LF- Lassa fever; N- total number of pregnant women included in analysis. a Adjusted for factors marginally significant in the univariate regression (crude p<0·25), except collinear terms. ND–Multivariable regression not performed as not of the variables satisfied the criteria for inclusion in a multivariable model. No factor or variable was significantly associated with [file pntd.0011209.s004.docx]

**A**

| Factor | Seropositive from baseline | β crude [95% CI] | P crude | β adjusted^a^ [95% CI] | P adjusted^a^ |
| --- | --- | --- | --- | --- | --- |
| N | 55 |  |  |  |  |
| Maternal age (years) | 55 | -0.009 [-0.03 -0.01] | 0.268 | — | — |
| Maternal LASV IgG conc | 55 | -0.076 [-0.15 -0.004] | 0.039 | -0.072 [-0.14 -0.001] | 0.052 |
| Parity | 55 | -0.0003 [-0.04 -0.04] | 0.987 | — | — |
| Fever during pregnancy |  |  |  |  |  |
| No | 44 | Baseline |  |  |  |
| Yes | 11 | 0.033 [-0.18 -0.25] | 0.759 | — | — |
| Positive history of LF |  |  |  |  |  |
| No | 54 | Baseline |  |  |  |
| Yes | 1 | -0.034 [-0.68 -0.62] | 0.916 | — | — |
| Malaria during pregnancy |  |  |  |  |  |
| No | 30 | Baseline |  |  |  |
| Yes | 25 | 0.113 [-0.09 - 0.32] | 0.273 | — | — |
| Hypertension |  |  |  |  |  |
| No | 53 | Baseline |  |  |  |
| Yes | 2 | -0.372 [-0.82 -0.08] | 0.105 | -0.321 [-0.75 -0.11] | 0.142 |
| Pregnancy-induced hypertension |  |  |  |  |  |
| No | 54 | Baseline |  |  |  |
| Yes | 1 | 0.112 [-0.54 -0.76] | 0.729 | — | — |
| Diabetes mellitus |  |  |  |  |  |
| No | 53 | Baseline |  |  |  |
| Yes | 2 | 0.166 [-0.29 -0.62] | 0.483 | — | — |
| HIV – infected |  |  |  |  |  |
| No | 49 | Baseline |  |  |  |
| Yes | 6 | 0.246 [-0.02 -0.52] | 0.074 | 0.192 [-0.07 – 0.44] | 0.149 |
| Gestational age (weeks) |  |  |  |  |  |
| ≥ 37 | 45 | baseline |  |  |  |
| < 37 | 3 | 0.195 [-0.20 -0.59] | 0.329 | — | — |
| Birthweight (Kg) |  |  |  |  |  |
| ≥ 2·5 | 50 | Baseline |  |  |  |
| < 2·5 | 4 | 0.009 [-0.33- 0.35] | 0.958 | — | — |
| Sex at birth |  |  |  |  |  |
| Male | 23 | Baseline |  |  |  |
| Female | 31 | 0.001 [-0.18 -0.18] | 0.995 | — | — |

**B**

| Factor | Seroconversion | β crude [95% CI] | p crude | β adjusted^a^ [95% CI] | p adjusted |
| --- | --- | --- | --- | --- | --- |
| N | 22 |  |  |  |  |
| Maternal age (years) | 22 | 0.0003 [-0.05 -0.06] | 0.912 | ND | ND |
| Maternal LASV IgG conc | 22 | -0.197 [-0.44 -0.04] | 0.103 | ND | ND |
| Parity | 22 | 0.0003 [-0.16 -0.16] | 0.997 | ND | ND |
| Fever during pregnancy |  |  |  |  |  |
| No | 16 | Baseline |  |  |  |
| Yes | 6 | -0.126 [-0.81 -0.55] | 0.704 | ND | ND |
| Positive history of LF |  |  |  |  |  |
| No | 21 | baseline |  |  |  |
| Yes | 1 | 0.054 [-1.41 -1.51] | 0.939 | ND | ND |
| Malaria during pregnancy |  |  |  |  |  |
| No | 13 | baseline |  |  |  |
| Yes | 9 | -0.306 [-0.91 -0.29] | 0.302 | ND | ND |
| Hypertension |  |  |  |  |  |
| No | 21 | Baseline |  |  |  |
| Yes | 1 | -0.464 [-1.91 -0.98] | 0.511 | ND | ND |
| Pregnancy-induced hypertension |  |  |  |  |  |
| No | 20 | Baseline |  |  |  |
| Yes | 2 | 0.048 [-1.01 -1.10] | 0.926 | ND | ND |
| HIV – infected |  |  |  |  |  |
| No | 21 | baseline |  |  |  |
| Yes | 1 | 0.001 [-1.46 -1.46] | 0.999 | ND | ND |
| Gestational age (weeks) |  |  |  |  |  |
| ≥ 37 | 20 | baseline |  |  |  |
| < 37 | 2 | -0.216 [-1.27 -0.84] | 0.674 | ND | ND |
| Birthweight (Kg) |  |  |  |  |  |
| ≥ 2·5 | 21 | baseline |  |  |  |
| < 2·5 | 1 | 0.053 [-1.41 – 1.51] | 0.940 | ND | ND |
| Sex at birth |  |  |  |  |  |
| Male | 11 | baseline |  |  |  |
| Female | 11 | 0.270 [-0.33 -0.87] | 0.355 | ND | ND |

**C**

| Factor | Seronegatives | β crude [95% CI] | P crude | β adjusted^a^ [95% CI] | P adjusted^a^ |
| --- | --- | --- | --- | --- | --- |
| N | 93 |  |  |  |  |
| Maternal age (years) | 93 | 0.054 [-0.16 -0.27] | 0.609 | ND | ND |
| Maternal LASV IgG conc | 93 | -0.457 [-2.87 -1.96] | 0.708 | ND | ND |
| Parity | 93 | 0.088 [-0.05 -0.23] | 0.268 | ND | ND |
| Fever during pregnancy |  |  |  |  |  |
| No | 76 | Baseline |  |  |  |
| Yes | 17 | -0.210 [-0.79 -0.37] | 0.471 | ND | ND |
| Positive history of LF |  |  |  |  |  |
| No | 91 | Baseline |  |  |  |
| Yes | 2 | -1.129 [-7.53 -5.28] | 0.727 | ND | ND |
| Malaria during pregnancy |  |  |  |  |  |
| No | 59 | Baseline |  |  |  |
| Yes | 34 | -0.205 [-0.73 -0.32] | 0.439 | ND | ND |
| Hypertension |  |  |  |  |  |
| No | 91 | Baseline |  |  |  |
| Yes | 2 | 1.231 [-5.18 -7.63] | 0.703 | ND | ND |
| Pregnancy-induced hypertension |  |  |  |  |  |
| No | 92 | Baseline |  |  |  |
| Yes | 1 | -1.098 [-10.1 -7.92] | 0.809 | ND | ND |
| Diabetes mellitus |  |  |  |  |  |
| No | 91 | Baseline |  |  |  |
| Yes | 2 | -1.129 [-7.54 -5.28] | 0.727 | ND | ND |
| HIV – infected |  |  |  |  |  |
| No | 92 | Baseline |  |  |  |
| Yes | 1 | -1.118 [-10.1 -7.89] | 0.806 | ND | ND |
| Gestational age (weeks) |  |  |  |  |  |
| ≥ 37 | 73 | baseline |  |  |  |
| < 37 | 7 | -1.346 [-5.16 -2.47] | 0.485 | ND | ND |
| Birthweight (Kg) |  |  |  |  |  |
| ≥ 2·5 | 87 | Baseline |  |  |  |
| < 2·5 | 6 | -1.124 [-4.90 -2.66] | 0.556 | ND | ND |
| Sex at birth |  |  |  |  |  |
| Male | 48 | Baseline |  |  |  |
| Female | 45 | -1.038 [-2.896 -0.819] | 0.279 | ND | ND |
