# Supplementary figures and images for: Transplacental transfer of Lassa IgG antibodies in pregnant women in Southern Nigeria: A prospective hospital-based cohort study
Source: PLoS Negl Trop Dis. 2023 Apr 13;17(4):e0011209. doi: 10.1371/journal.pntd.0011209 (PMC10129015; doi:10.1371/journal.pntd.0011209)

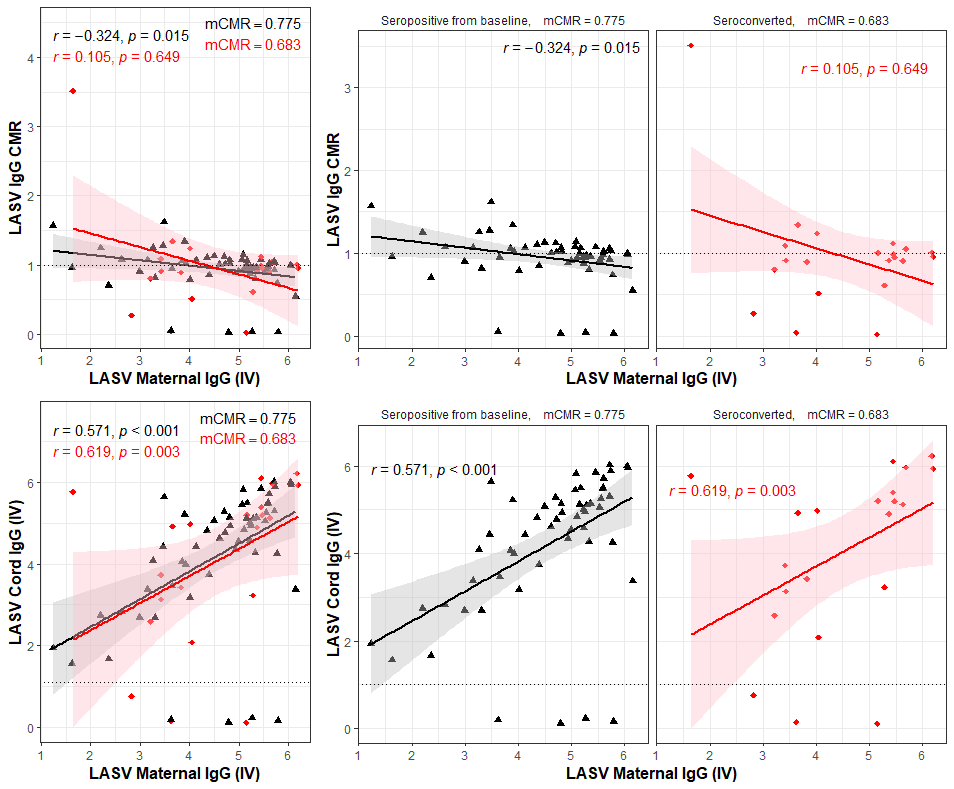

Supplement: S1 Fig — Note: The figure evaluates the relationship between maternal LASV IgG concentration and placental transfer measured as the CMR and the relationship between maternal LASV IgG with cord LASV IgG concentration while accounting for seroconversion. Black indicates seropositive from baseline and red indicates seroconversion. The dotted black horizontal line indicates the line of efficient transfer (CMR = 1); IV- index value, an arbitrary antibody concentration unit based on manufacturer’s guide; mCMR- median cord maternal ratio; black and red lines are regression lines with the corresponding 95% confidence interval fitted to the regression line. (TIF) [file pntd.0011209.s006.tif]
